# α-Tocopheryl Succinate-Based Polymeric Nanoparticles for the Treatment of Head and Neck Squamous Cell Carcinoma

**DOI:** 10.3390/biom8030097

**Published:** 2018-09-19

**Authors:** Carolina Sánchez-Rodríguez, Raquel Palao-Suay, Laura Rodrigáñez, María Rosa Aguilar, Sergio Martín-Saldaña, Julio San Román, Ricardo Sanz-Fernández

**Affiliations:** 1 Faculty of Biomedical and Health Sciences, Universidad Europea de Madrid, Calle del Tajo S/N, Villaviciosa de Odón, 28670 Madrid, Spain; carolina.sanchez2@universidadeuropea.es (C.S.-R.); ricardosanz.orl@gmail.com (R.S.-F.); 2 Group of Biomaterials, Department of Polymeric Nanomaterials and Biomaterials, Institute of Polymer Science and Technology, ICTP-CSIC, C/Juan de la Cierva, 3, 28006 Madrid, Spain; rpsuay@ictp.csic.es (R.P.-S.); serg_1112@hotmail.com (S.M.-S.); jsroman@ictp.csic.es (J.S.R.); 3 Networking Biomedical Research Centre in Bioengineering, Biomaterials, and Nanomedicine, CIBER-BBN, Av. Monforte de Lemos, 3-5, 28029 Madrid, Spain; 4 Department of Otolaryngology, Hospital Universitario de Getafe, Carretera de Toledo, km 12,500, 28905 Getafe (Madrid), Spain; laurarodriganez@gmail.com

**Keywords:** α-tocopheryl succinate, polymeric nanoparticle, angiogenesis, migration, hypopharynx carcinoma squamous cells, proliferating endothelial cells

## Abstract

The aim of this work is to study, in an in vitro head and neck squamous cell carcinomas model the anti-angiogenic and anti-migratory properties of self-assembled polymeric nanoparticles (NPs) with demonstrated selective anticancer activity. The NPs are based on α-tocopheryl succinate (α-TOS) encapsulated in the hydrophobic core of the NPs. We analyzed the effect of the newly synthetized α-TOS-loaded NPs in proliferating endothelial cells and hypopharynx carcinoma squamous cells and measured markers of angiogenesis, apoptosis and reactive oxygen species (ROS). α-TOS-loaded NPs suppressed angiogenesis by inducing accumulation of ROS and inducing apoptosis of proliferating endothelial cells. These NPs also decrease the number and quality of capillary-like tubes in an in vitro three-dimensional (3D) experiment, decrease the production of the pro-angiogenic vascular endothelial growth factor and down-regulate the expression of its receptor. The anti-migratory efficacy of α-TOS is corroborated in hypopharynx carcinoma cells by decreasing the secretion of matrix metalloproteases 2 and 9 (MMP-2 and MMP-9) and inhibiting cell migration. These results confirm that α-TOS-based NPs not only present anticancer properties, but also antiangiogenic properties, therefore making them promising candidates for multi-active combinatorial anticancer therapy.

## 1. Introduction

Head and neck squamous cell carcinomas (HNSCC) are highly angiogenic, and their vasculature expresses numerous angiogenic cytokines, including vascular endothelial growth factor (VEGF), interleukin-1a and fibroblast growth factor (FGF) [1,2,3]. Moreover, overexpression of matrix metalloproteinases (MMP) in HNSCC is frequently observed and has been linked with tumor invasion, metastasis and poor survival [4]. Therefore, antiangiogenic compounds are being explored in HNSCC cancer therapies, as they suppress the process of neovascularization, avoiding the tumor growth [5].

Highly specific inhibitors of both VEGF (e.g., bevacizumab) and its receptor VEGFR (e.g., sorafenib) have been developed. Some of them have entered human clinical trials but have not produced sufficient activity to warrant approval. The first to receive the approval of the Food and Drug Administration (FDA) in 2004 was bevacizumab for metastasic colorectal cancer, followed by sutinib and sorafenib [6]. However, none of these are being used successfully in the treatment of HNSCC, which has recently been studied in a phase II trial with docetaxel [7]. Further studies are needed to find the ideal antiangiogenic therapy for HNSCC.

The anticancer and antiangiogenic properties of α-tocopheryl succinate (α-TOS) have been extensively demonstrated [8,9]. It is a well-known mitochondrially targeted anticancer compound (mitocan) that induces the accumulation of reactive oxygen species (ROS) and modifies mitochondrial permeability by shifting the balance of pro- and anti-apoptotic Bcl-2 family proteins, leading to the activation of the mitochondrial apoptotic pathway [10,11,12]. Moreover, α-TOS inhibits angiogenesis by the selective induction of apoptosis in proliferating endothelial cells (ECs), while being non-toxic to arrested ECs forming the inner lining of normal blood vessels [8,13].

Despite all these properties, the hydrophobic nature of the drug, which significantly reduces its bioavailability and therapeutic activity, is the main obstacle for the successful application of α-TOS-based clinical treatments. Our group has incorporated this molecule into nanovehicles of appropriate hydrodynamic properties in order to improve the biological activity of α-TOS [14,15,16,17]. In this sense, the methacrylic derivative of α-TOS was synthesized and copolymerized forming amphiphilic macromolecules that were able to self-assemble in aqueous media forming surfactant-free nanoparticles (NPs). The hydrophilic segment of the copolymers was mainly based on *N*-vinyl pyrrolidone (VP), and the hydrophobic segment mostly incorporated MTOS (poly(VP-*co*-MTOS)(89:11)) [14,17]. Unloaded NPs (NP-0) presented in vitro anticancer activity in different cancerous cell lines and had the capacity to selectively reduce cell viability of proliferating ECs. Moreover, α-TOS encapsulation in the inner core of the NPs (NP-10) enhanced the pro-apoptotic activity of these nanoassemblies on hypopharynx carcinoma squamous cells (FaDu) [17]. For these reasons, the aim of this work is to analyze the in vitro anti-angiogenic and anti-invasive effect of these recently synthetized α-TOS-based NPs (NP-0 and NP-10).

## 2. Materials and Methods

### 2.1. Synthesis of the Copolymers and Nanoparticles

The synthesis of the methacrylic derivative of α-TOS (MTOS), the copolymer poly(VP-*co*-MTOS) with a copolymer molar composition of VP:MTOS 89:11 (poly(VP-*co*-MTOS)(89:11)), and the nanoparticles used in this work were recently described by our group [14,17]. Briefly, MTOS was obtained by the condensation reaction of α-tocopherol and mono-(2-(methacryloyloxy)ethyl succinate (1.4 equiv) in the presence of 4-dimethylaminopyridine (DMAP, 0.1 equiv) and *N*,*N*′-dicyclohexylcarbodiimide (DCC, 1.5 equiv) in an ice bath under nitrogen atmosphere. Poly(VP-*co*-MTOS)(89:11) was obtained by free radical copolymerization of both monomers (total monomer concentration 1 M; feed molar composition VP:MTOS 85:15) in dioxane at 60 °C, for 24 h. The NP formulations (NP-0 and NP-10) were obtained by nanoprecipitation of the copolymer solution in dioxane (10 mg/mL) added drop by drop over an aqueous phase (phosphate buffered saline, PBS, 0.01 M, pH 7.4). Final concentration of the nanoparticles was 2 mg/mL. Hydrodynamic diameter (D_h_) and polydispersity (PDI) were determined by dynamic light scattering (DLS), zeta potential (ζ) by laser doppler electrophoresis, and encapsulation efficiency (EE) by ultraviolet (UV) spectroscopy. These characterization results, including hydrodynamic properties, encapsulation efficiency, scanning electron microscopy (SEM), transmission electron microscopy (TEM), atomic force microscopy (AFM) and confocal fluorescence of coumarin-6-loaded NPs were presented in previous publications [14,17]. Table 1 summarizes the most relevant properties of the NPs.

In vitro release of α-TOS was carried out by dialysis of 5 mL of NP-0 or NP-10 against 10 mL of PBS with esterases (15 U/mL) for 15 days at 37 °C. One milliliter aliquots were withdrawn at different time points and stored for quantitative analysis of α-TOS, and the same volume of PBS was replenished; however, α-TOS was not detected by either high performance liquid chromatography (HPLC) or gas chromatography (GC). High performance liquid chromatography analysis was performed on a C18-column (4.6 mm × 250 mm, Agela Technologies, Torrance, CA, USA) at 30 °C. Different mobile phases pumped at a rate of 1 mL/min were used for the analysis: acetonitrile:milliQ water (80:20, *v*/*v*); methanol:milliQ water (98:2 *v*/*v*); acetonitrile:methanol (60:40 *v*/*v*); methanol:milliQ water (90:10 *v*/*v*) with 0.1% of trifluoroacetic acid. The UV detector was set at λ_abs_ = 285 nm. The experiment was carried out in triplicates. α-Tocopheryl succinate (α-TOS) release was not detected with none of the mobile phases tested. Additionally, GC was also tested to measure α-TOS release with similar results. The separation of the additives was carried out using a Hewlett Packard 6890 HRGC high resolution gas chromatograph (Hewlett Packard, Palo Alto, CA, USA) equipped with a 5973 mass spectrometer detector. ADB-5 fused silica capillary column (30 m × 250 µm and 0.25 µm film thickness) was used. The carrier gas was helium pumped at a rate of 1 mL/min, with a trend of temperature (10 °C/min) from 80 to 300 °C [18].

### 2.2. Cell Culture and Treatment

FaDu cells from American Type Culture Collection (ATCC) were cultured in Dulbecco’s modified Eagle’s medium (DMEM) containing 10% fetal bovine serum (FBS, Lonza, Verviers, Belgium) and 1% penicillin/streptomycin (Sigma-Aldrich, San Luis, MO, USA). Human aortic endothelial cells (HAEC; Lonza) were cultured in Endothelial Cell Growth Medium-2 EGM-2 MV Bullet kit medium (Lonza). Cell cultures were incubated at 37 °C and 5% CO_2_.

Human aortic endothelial cells (HAEC) were used because these cells, unlike the primary endothelial cells with limited life span (human umbilical vein endothelial cells, HUEVEC, usually four to five cell cycle transitions), are guaranteed through 15 population doublings, express CD31/105, von Williebrand Factor VIII, and are positive for acetyated low density lipoprotein uptake; as well as formation of tubes in a three-dimensional setting and persistent arrest. Conventionally, HUVECs and HAECs are representative endothelial cell types isolated from human blood vessels, and both cell types show similar cellular characteristics and morphology [19,20].

The experiments with HAEC were carried out under conditions of proliferation (50% confluency) or arrest (100% confluency). The study of the antiangiogenic activity at different confluences was described in previous published studies of our group, Raquel Palao-Suay et al. 2015 [14] and Dong et al. 2007 [8].

Nanoparticle-0 (NP-0) and NP-10 were used at 1 and 0.25 mg/mL, respectively. As previously demonstrated, NP-10 at a concentration of 0.25 mg/mL was the highest concentration of the NPs loaded with the highest concentration of α-TOS that presented selectivity against FaDu cancer cells and was not toxic to quiescent endothelial cells. Nanoparticle-0 (NP-0) presented lower biological activity than NP-10, and was therefore added at a higher concentration (1 mg/mL) to demonstrate the intrinsic biological activity of the polymeric nanovehicle [17]. Cells were treated with NPs for 24 h. In some experiments, HAEC were pre-treated with 900 × 10^−9^ M mitochondrially targeted coenzyme Q (MitoQ) for 1 h.

### 2.3. Cell Viability Assay

Cell viability in the presence of NP-0 and NP-10 was measured using 3-(4,5-dimethylthiazol-2-yl)-2,5-diphenyltetrazolium bromide (MTT) assay. Cells were seeded in 96-well plates at 2500 cells/well (FaDu) and 5000 cells/well (HAEC). After 24 h of incubation, the medium was replaced with the corresponding NPs (50 µL of the NPs suspension and 50 µL of completed medium) and the plates were incubated for 24 h.

### 2.4. Reactive Species Quantification (Total ROS)

Total ROS free radical activity was measured by the OxiSelect^TM^ In Vitro ROS Assay Kit, which employs the specific ROS probe 2′,7′-dichlorodihydrofluorescein DiOxyQ (DCFH-DiOxyQ, Cell Biolabs, San Diego, CA, USA). Supernatants of HAEC cultures, after being in contact with PBS, NP-0 or NP-10 (50 μL each), were added to the wells with 50 μL of the catalyst (diluted in PBS at 1:250). Afterwards, 100 μL of DCFH probe was added to each well. The samples were measured fluorometrically (480 nm excitation/530 nm emission) and free radical content was determined by comparison with the predetermined 2′,7′-dichlorofluorescein DCF standard curve.

### 2.5. Superoxide Anion Detection

Superoxide generation was qualitatively determined using red fluorescent-labeled dihydroethidium (DHE; Calbiochem, San Francisco, CA, USA). HAEC were fixed with 4% paraformaldehyde (PFA), blocked and incubated for 90 min at 37 °C with DHE (4 µmol/L). After washing with 0.1% Triton X-100 in PBS, cells were visualized in an Olympus BX51 fluorescence microscope (Olympus, Southend-on-Sea, England).

### 2.6. Western Blot

Briefly, blots were probed with polyclonal antibodies against VEGFR (1/100), active caspase-3 (1/100), MMP-2 and MMP-9 (1/100) and β-actin (1/2000, used as a loading control), all from Abcam. After washing, blots were probed with a rabbit peroxidase-conjugated secondary antibody (1/10,000; Abcam) and immunoreactive bands were visualized with Supersignal West Pico Chemiluminescent substrate (ThermoFisher Scientific, Waltham, MA, USA and quantified by densitometry using AlphaFaseFC software ChemiImager^TM^5500 (Alpha Innotech Corporation, San Leandro, CA, USA).

### 2.7. Annexin V Detection by Indirect Immunofluorescence

FaDu cells were fixed in 4% PFA and washed with PBS. Cells were incubated for 1 h in 0.3% Triton X-100/PBS, blocked for 1 h at 37 °C and incubated overnight at 4 °C with a rabbit polyclonal anti-Annexin V antibody (1/75; Abcam). Following washing in PBS, cells were incubated with an Alexa Fluor 546-conjugated goat anti-rabbit antibody (1/250; Molecular Probes, Eugene, OR, USA) for 45 min at 37 °C. After further PBS washes, cells were stained for 5 min with 300 nM of 4′,6-diamidino-2-phenylindole dihydrochloride (DAPI; Sigma-Aldrich). Cells were scored for fluorescence using an Olympus BX51 fluorescence microscope (Olympus) and the specificity was evaluated by omission of the primary antibody.

### 2.8. Cytokine Quantification

Cell-free conditioned medium from each treatment group was collected and used to measure the levels of VEGF using commercial enzyme-linked immunosorbent assay (ELISA) kit (Quantikine, R&D systems, Minneapolis, MN, USA). The colorimetric reaction was measured at 450 nm using a microplate reader (GENios Plus, TECAN, Männedorf, Switzerland) and the readings were converted to pg/mL using standard curve obtained with recombinant cytokine.

### 2.9. In Vitro Angiogenesis

Human aortic endothelial cells (HAEC) seeded into Matrigel-precoated wells was examined in the presence of PBS (control group), NP-0, or NP-10. At the indicated periods, five randomly selected fields of view were photographed per well using an Olympus CKX41 inverted microscope. The number of branch points (nodes), mesh-like circles (circles) and tube-like structures (tubes) were quantified using the angiogenesis analyzer for ImageJ (NIH). Each sample was tested in duplicate from four independent experiments.

### 2.10. Wound Healing Assay

Cells migration in the presence of the NPs was evaluated using CytoSelect™ kit (Cell Biolabs). In a 24-well plate, inserts were placed in each well in contact to the bottom. A cell suspension containing 0.5 × 10^6^ FaDu cells/mL containing 10% FBS was created. 500 μL of cell suspension was added to each well and incubated overnight. The inserts were then removed and the wells washed with media and incubated for another 24 h. Cells were fixed and visualized using an Olympus CKX41 inverted microscope. Images were automatically analyzed using TScratch software (CSElab, version 1.0, Zurich, Switzerland).

### 2.11. Statistical Analysis

Results were expressed as mean ± standard deviation. Statistical significance (*p* < 0.05) was evaluated using the analysis of variance (ANOVA, Tukey test) by Origin 9 (OriginLab, version 9, Northampton, MA, USA).

### 2.12. Ethics Approval and Consent to Participate 

Not applicable. Our study did not involve human or animal research.

## 3. Results

### 3.1. α-Tocopheryl Succinate In Vitro Release

α-Tocopheryl succinate (α-TOS) was not detected by HPLC or GC under the conditions previously described. However, in the in vitro biological studies, the NPs were taken up by the cells, and the biological effect was observed (induction of apoptosis of proliferating endothelial cells and cancer cells). Therefore, we assume that not only was the α-TOS cargo released (as demonstrated by the higher biological activity of NP-10 compared to NP-0), but the copolymer also presented biological activity (as demonstrated by the results obtained with NP-0) [14,17].

### 3.2. Reduction of Endothelial Cell Viability

**Figure 1:**
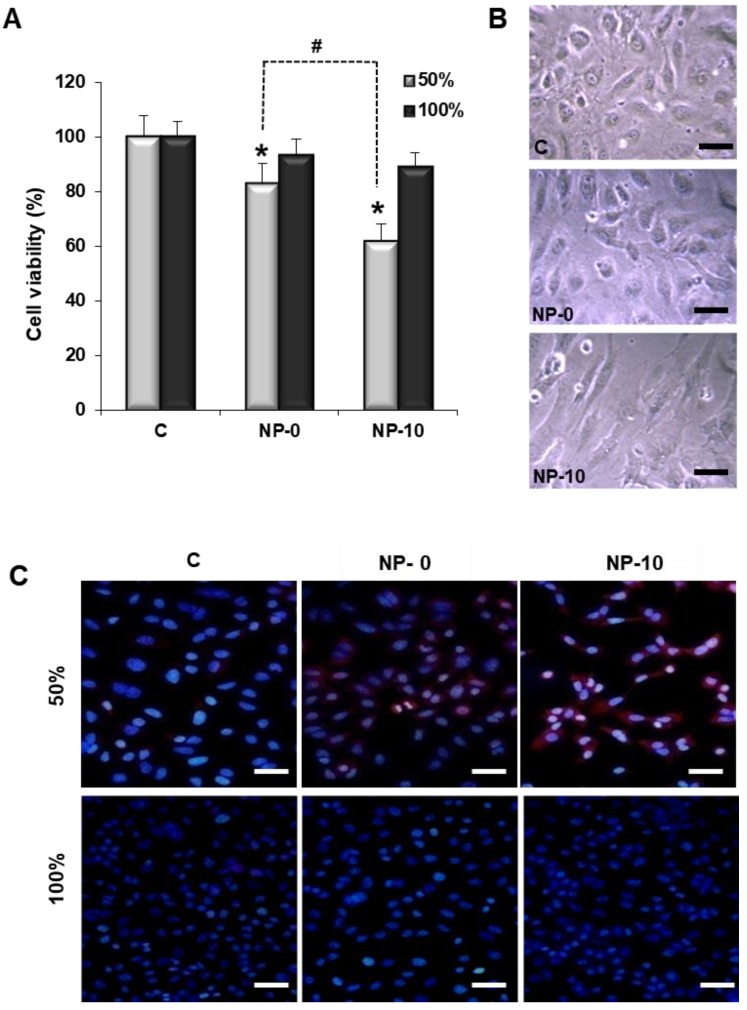
Inhibition of proliferation of human aortic endothelial cells (HAEC) exposed to phosphate buffered saline (PBS) (C–control), NP-0, or NP-10 for 24 h. (**A**) HAEC viability of cultures at 50% and 100% confluency. The diagrams include the mean, the standard deviation (*n* = 4), and the analysis of variance (ANOVA) results (* *p* < 0.05 and # *p* < 0.05 statistically significant difference with control and NP-10, respectively). (**B**) Representative optical micrographs of proliferating HAEC at 50% confluency (×40). Scale bar 100 µm. (**C**) Fluorescence micrographs of representative immunostaining of Annexin V-Alexa Fluor546 conjugate in HAEC cells (red) and 4′,6-diamidino-2-phenylindole dihydrochloride (DAPI) (blue) (×40). Scale bar 100 µm.

Cell viability of HAEC in the presence of NP-0 and NP-10 was studied at high proliferative status (50% confluency), and non-proliferative status (100% confluency, at which the majority of the cells are growth arrested in G0). HAEC viability significantly decreased to 80% and 60% in the presence of NP-0 and NP-10, respectively, in comparison with the control group in proliferating cells. The observed difference between NP-0 and NP-10 reached statistical significance, however, there were no significant differences between groups in confluent cells (Figure 1A). Gross inspection of HAEC by optical microscopy showed that in the control and NP-0 groups, HAEC exhibited typical endothelial morphological features and were polyhedral in shape, slightly elongated, refractile with an elliptical core, connected, and with abundant intracytoplasmic vesicles. By contrast, NP-10 treatment was associated with dramatic changes to HAEC morphology, including elongation and spreading of the cell cytoplasm together with a qualitative decrease in the number of cells (Figure 1B).

### 3.3. Apoptosis Induction and Oxidative Stress in Endothelial Cells

**Figure 2:**
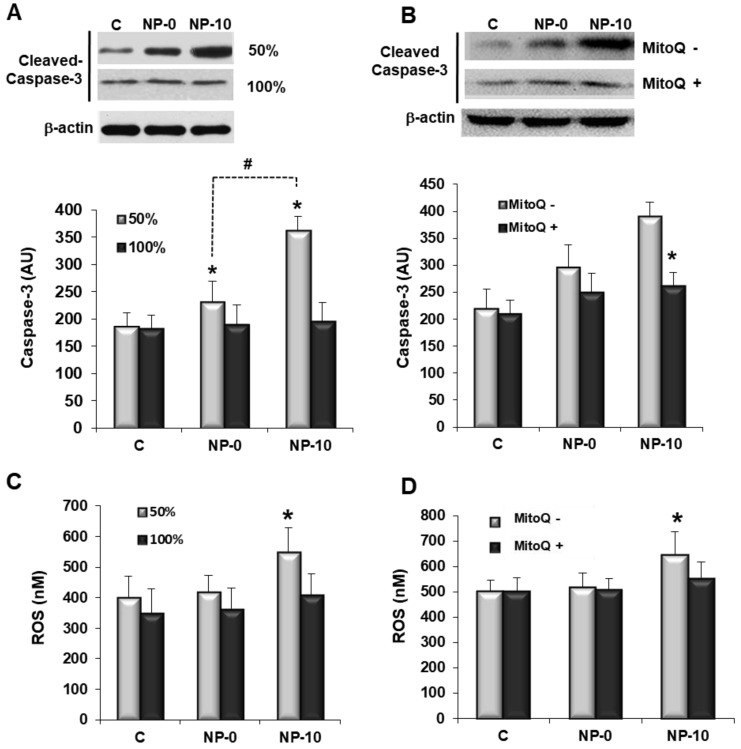

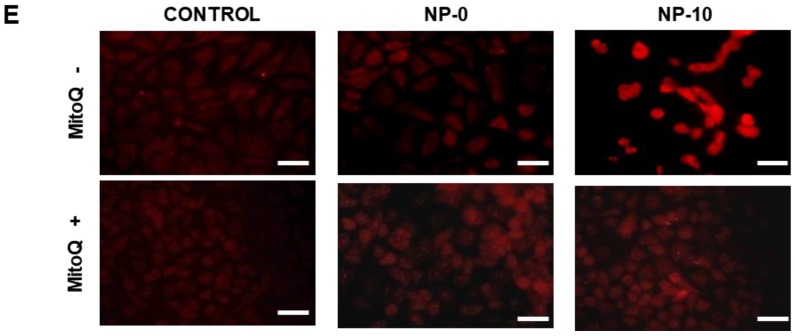
Apoptosis induction and oxidative stress in HAEC after treatment with PBS (control), NP-0 or NP-10 for 24 h. (**A**,**B**) Caspase-3 expression (in arbitrary units, AU) and (**C**,**D**) total reactive oxidative species (ROS) concentration of HAEC cultures at 50% and 100% confluency. Human aortic endothelial cells cultures at 50% confluency in the presence or absence of MitoQ (**B**,**D**). The diagrams include the mean, the standard deviation (*n* = 6), and the ANOVA results (* *p* < 0.05 and # *p* < 0.05 statistically significant difference with control and NP-10, respectively). (**E**) Representative images of HAEC incubated with dihydroethidium DHE dye (×40). Scale bar 100 µm.

Apoptosis induction by NPs as a function of the HAEC proliferative status was qualitatively analyzed by immunocytochemistry using Annexin V staining of DAPI counter-stained cells. Fluorescence micrographs revealed a red fluorescence increase in HAEC cells at 50% confluency incubated with NP-10, indicating an increase of apoptosis (Figure 1C). Apoptosis was also quantitatively studied by measuring the levels of the apoptosis effector protein caspase-3 by western blotting of HAEC cell lysates. After both NP treatments, highly proliferative HAEC cells had significantly higher levels of active caspase-3 expression in comparison to the control group. Moreover, statistically significant differences between the levels of caspase-3 for NP-0 and NP-10 groups were found, with caspase-3 activity being the highest for NP-10. However, cells at 100% confluence did not show significant differences in active caspase-3 expression in the three treatment groups (Figure 2A).

Total ROS free radical activity was quantified using a DCFH probe. The increment of ROS concentration was remarkable after the treatment of proliferative HAEC with NP-10 group, specifically increasing above 138% in comparison to the control. In contrast, there was hardly any detectable increase in the level of ROS in confluent cells exposed to α-TOS-based NPs (Figure 2C). To study the ROS-apoptosis relation, proliferative HAEC were exposed to the NPs in the presence of MitoQ [21]. The data in Figure 2B,D show that MitoQ effectively suppressed both ROS accumulation and caspase-3 expression in proliferating HAEC.

### 3.4. In Vitro Antiangiogenic Activity of Endothelial Cells

**Figure 3:**
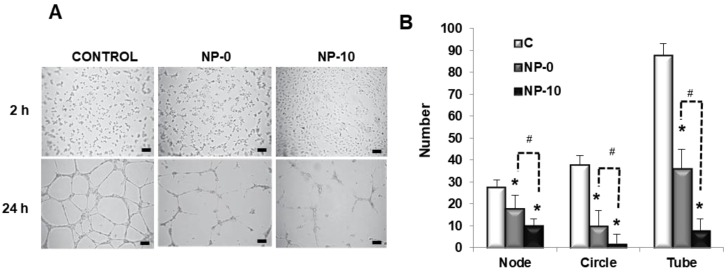

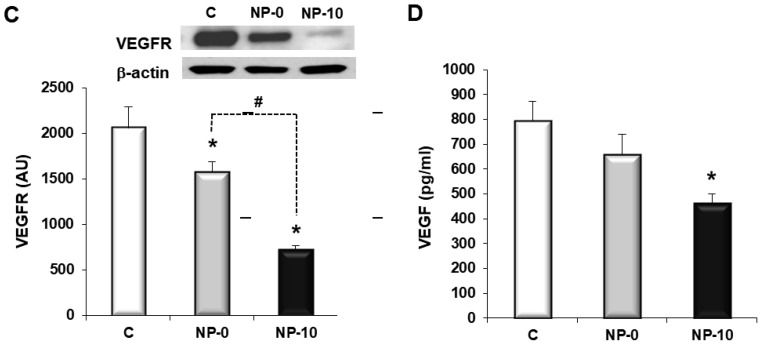
In vitro antiangiogenic activity of HAEC exposed to PBS (control), NP-0, or NP-10 for 24 h. (**A**) Representative contrast-phase images of HAEC three-dimensional (3D) cultures in Matrigel (×40). Scale bar 100 µm. (**B**) Number of formed nodes, circles and tubes of HAEC cultures. (**C**) VEGFR expression and (**D**) VEGF levels after NPs treatment. The diagrams include the mean, the standard deviation (*n* = 6), and the ANOVA results (* *p* < 0.05 and # *p* < 0.05 statistically significant difference with control and NP-10, respectively).

The antiangiogenic activity of self-assembled NPs was analyzed by the evaluation of tube-like forming capacity of HAEC in a three-dimensional setting. In the absence of NPs, HAEC formed capillary-like structures, branched nodes and mesh-like circles, indicative of the primary and interim stages of angiogenesis. However, in the presence of the α-TOS-based NPs, cells did not assemble or interconnect, significantly decreasing the number of nodes and circles (Figure 3A). Moreover, the evaluation of the number of tubes, indicative of late-phase angiogenesis, revealed an inhibitory effect of NP-0 and NP-10, with 86 tubes per field for control cells, 35 for cells exposed to NP-0, and less than seven tubes for cells exposed to NP-10 (Figure 3B).

To deepen the potential of self-assembled NPs as targets to develop anti-angiogenic therapies, VEGFR expression and VEGF levels were quantified after the NP-0 and NP-10 treatments by Western blot analysis in protein extracts from proliferative HAEC cell cultures lysates (Figure 3C,D). In fact, results showed a decrease in VEGFR expression and VEGF levels when HAEC were treated with NP-0 and NP-10. Particularly, VEGFR expression was significantly downregulated by 67% and 52% with respect to the control and NP-0, respectively, using α-TOS loaded NPs (NP-10).

### 3.5. Inhibition of Matrix Metalloproteinases Expression and Cell Migration of Hypopharynx Carcinoma Cells

**Figure 4:**
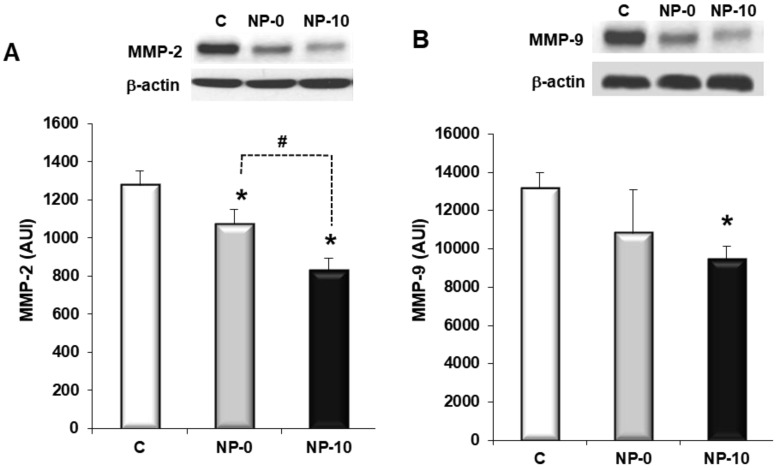

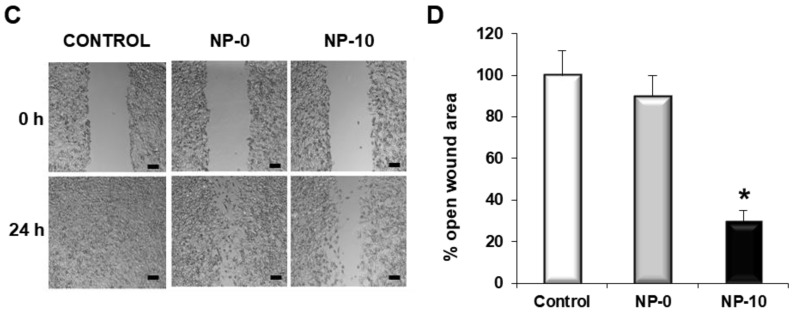
Inhibition of matrix metalloproteinases (MMP) expression and cell migration (in arbitrary units, AU) of FaDu cells exposed to PBS (control), NP-0 or NP-10 for 24 h. (**A**,**B**) Representative Western blots of MMP-2 (**A**) and MMP-9 (**B**) protein expression levels and densitometric analysis (*n* = 6). (**C**) Representative phase-contrast micrographs (×40) of cell migration. Scale bar 100 µm. (**D**) % open wound area after culture for 24 h. The diagrams include the mean, the standard deviation (*n* = 6), and the ANOVA results (* *p* < 0.05 and # *p* < 0.05 statistically significant difference with control and NP-10, respectively).

Both NP-0 and NP-10 showed a significant decrease of MMP-2 when compared with the control (Figure 4A). The reduction of MMP-9 was statistically significant in the NP-10 group in comparison with the control. NP-0 decreased MMP-9 levels but did not reach statistical significance when compared with the control (Figure 4B). In addition, NP-0 allowed FaDu to proliferate and migrate through the denuded region; however, NP-10 efficiently prevented this process (Figure 4C,D) in the wound healing assay.

## 4. Discussion

The tumor microenviroment is sustained and maintained by the production of growth factors, cytokines and proteases that stimulate tumor cell proliferation and migration, and trigger angiogenesis. Tumor angiogenesis begins with vasodilation and increase permeability of existing vessels in response to VEGF, which is accompaigned by the loss of perycites. Meanwhile, many secreted matrix-degrading enzymes (e.g., MMP), dissolve the basement membrane and remodel the extracellular matrix [22,23] to facilitate cell migration and self-assembling to form new blood vessels that irrigate and provide nutrients to the growing tumor. The aim of this work was to study whether α-TOS-based NPs also present the same anti-angiogenic and anti-invasive properties.

Anticancer drugs can exert their activity via several modes of action. Most agents act by direct killing of malignant cells. However, an intriguing option to promote suppression of tumors is to starve them of energy and oxygen, that is, to suppress the process of neovascularization of tumors by inhibiting angiogenesis [5]. The process of neovascularization is based either on the sprouting of new blood vessels from pre-existing vessels [24], or on recruitment and differentiation of endothelial progenitor cells [25]. The formation of new capillaries through sprouting requires diverse steps: firstly, endothelial cell activation and the breakdown of the basement membrane, followed by proliferation, migration and finally the tube formation of the endothelial cells [26]. The tube-like forming capacity of HAEC cells after NP treatment allowed the quantification of nodes, circles and tubes as parameters of the gradual regeneration process of angiogenesis, representing the primary, interim and later phases, respectively [27]. Both, NP-0 and NP-10 inhibited the endothelial tube-like formation, and reduced the number of nodes and circles in 3D HAEC cultures (Figure 3A,B). This was also observed with other vitamin E analogs such a γ-tocotrienol that inhibited migration and new blood vessel formation of HUVEC cells in a dose-dependent manner [8,26].

Another possibility to suppress angiogenesis is the induction of apoptosis selectively in proliferating ECs. Cell viability of proliferating HAEC was selectively reduced by NP-0 (1.00 mg/mL) and NP-10 (0.25 mg/mL), which demonstrated that α-TOS incorporated chemically and physically in the NPs is active during the time of the experiment, 24 h (Figure 1A,B). Cell viability was reduced due to the selective induction of oxidative stress and the accumulation of ROS (Figure 2C) that triggers apoptosis, as Annexin-V and caspase-3 highly increased in proliferating HAEC cultures in the presence of the NPs (Figure 1C and Figure 2A, respectively). Actually, NP-0 and NP-10 activated the mitochondrial apoptotic pathway (intrinsic pathway), as ROS accumulation and apoptosis induction in proliferating HAEC were inhibited in the presence of the mitochondrially targeted antioxidant MitoQ (Figure 2B,D,E) [21]. This selectivity is due to a specific up-regulation of the anti-oxidant systems in the arrested ECs, (e.g., manganese superoxide dismutase, MnSOD), which avoids the accumulation of ROS and the induction of oxidative stress [8,13]. These results are in agreement with those obtained by Dong et al., which demonstrated that the induction of apoptosis by targeting the mitochondria of proliferating ECs is a plausible factor by which α-TOS inhibits angiogenesis [8]. Moreover, these results suggest that agents such as α-TOS will efficiently kill angiogenic ECs of tumorigenic blood vessels while being nontoxic to the arrested ECs of normal blood vessels [28].

It has been reported that angiogenesis can be suppressed by interfering with processes essential for its promotion and maintenance, in particular, disrupting paracrine signaling between tumor cells and endothelial cells (ECs) [29]. In addition to apoptosis as a mechanism to explain the antiangiogenic activity of α-TOS-based NPs, the effect of these nanoassemblies on the expression of proangiogenic factors contributes to the promotion of this activity. Specifically, VEGF is considered the major mediator of endothelial cell proliferation and migration, activating angiogenic gene expression in that enhances vascular permeability [30]. In this way, different authors have demonstrated that α-TOS inhibits VEGF and fibroblast growth factor 2 FGF2 of breast cancer and mesothelioma cells [31,32,33]. Additionally, other vitamin E analogs, such as different isomers of tocotrienol, down-regulated phospho-VEGFR-2 levels through their inhibitory action on Wnt signaling [34]. Recently, we reported results demonstrating that α-TOS-based NPs reduced VEGF levels in FaDu cultures [17]. Moreover, this behavior was also observed in HAEC cells, significantly improving with the load of α-TOS into the NPs (Figure 3C,D). These results open the possiblity of using these nanoassemblies for dual-anticancer and anti-angiogenic therapies. In this sense, VEGF provides an excellent target for therapeutic intervention, acting as a point of integration of a wide range of uppstream and downstream signals. Moreover, VEGF acts directly on the endothelial cells, which are more genetically stable in comparison to cancer cells [35].

Another attractive strategy to develop antiangiogenic therapies is related to the inhibition of MMPs, which are secreted by tumor cells to degrade the extracellular matrix, stimulating cancer cell growth, migration, invasion, angiogenesis and metastasis formation. Metastasis formation in tumors is a complex process, with the extracellular matrix being the first barrier to avoiding tumor growth. Among MMPs, MMP-2 and MMP-9 upregulation has been associated with invasive tumors, and they are considered to play an important role in invasion and metastasis formation of cancer cells [36]. Unfortunately, their clinical utility has not succeeded due to their adverse effects, as well as to their paradoxical metastasis-promoting effect [37,38]. Zhang et al. demonstrated that α-TOS had an inhibitory effect on infiltration of metastasis formation of prostate cancer, involving the reduction of MMP-9 activity [33]. In our case, the data shown in Figure 4 indicate that MMP-2 and MMP-9 expression was downregulated, and cell migration was inhibited by NP-0 and NP-10 in FaDu cells. Additionally, the load of additional α-TOS in the core of the self-assembled NPs significantly improved the downregulation of MMPs, suggesting that the synthesized nanovehicles can enhance the anti-angiogenic activity of α-TOS.

## 5. Conclusions

The NPs described in this work not only induce apoptosis of tumor cells, but also of proliferating endothelial cells, whilst being harmless to non-tumoral cells and quiescent endothelial cells present in normal vasculature. Additionally, α-TOS-based NPs decrease the production of the pro-angiogenic growth factor VEGF and downregulate the expression of its receptor VEGFR. The secretion of proteases that degrade the extra-cellular matrix (MMP-2 and MMP-9) and migration of FaDu cells is also diminished. Due to these anti-angiogenic and anti-invasive properties, α-TOS-based NPs constitute a promising anticancer compound to target head and neck squamous tumors at multiple levels. Moreover, α-TOS-based NPs with an additional α-TOS cargo present not only an enhanced anti-cancer activity, but also an enhanced anti-angiogenic activity, which could represent an important advantage in the new antitumor therapies.

## Figures and Tables

**Table 1:** Hydrodynamic diameter (D_h_), polydispersity (PDI), zeta potential (ζ), and encapsulation efficiency (EE) of nanoparticle (NPs) formulations. α-Tocopheryl succinate (α-TOS).

NP	Drug	Load (% *w*/*w*)	D_h_ (nm)	PDI	ζ (mV)	EE (%)
NP-0	-	-	134.3 ± 9.2	0.128 ± 0.022	−3.0 ± 0.2	-
NP-10	α-TOS	10	164.3 ± 4.9	0.177 ± 0.042	−18.2 ± 0.6	72
